# Regulation of Intestinal Inflammation by Walnut-Derived Bioactive Compounds

**DOI:** 10.3390/nu16162643

**Published:** 2024-08-10

**Authors:** Kexin Dai, Neel Agarwal, Alexander Rodriguez-Palacios, Abigail Raffner Basson

**Affiliations:** 1 Department of Biology, Case Western Reserve University School of Medicine, Cleveland, OH 44106-4909, USA; kxd389@case.edu; 2 Department of Nutrition, Case Western Reserve University School of Medicine, Cleveland, OH 44106-4909, USA; 3 Department of Biochemistry, Case Western Reserve University School of Medicine, Cleveland, OH 44106-4909, USA; nxa357@case.edu; 4 Germfree Mouse Models Core, Case Western Reserve University School of Medicine, Cleveland, OH 44106-4909, USA; axr503@case.edu; 5 University Hospitals Research and Education Institute, University Hospitals Cleveland Medical Center, Cleveland, OH 44106-4909, USA; 6 Department of Molecular Biology and Microbiology, Case Western Reserve University School of Medicine, Cleveland, OH 44106-4909, USA; 7 Division of Gastroenterology and Liver Disease, Case Western Reserve University School of Medicine, Cleveland, OH 44106-4909, USA; 8 Digestive Health Research Institute, Case Western Reserve University School of Medicine, Cleveland, OH 44106-4909, USA

**Keywords:** walnuts, inflammatory bowel disease (IBD), mechanisms, bioactive compounds, inflammation

## Abstract

Walnuts (*Juglans regia* L.) have shown promising effects in terms of ameliorating inflammatory bowel disease (IBD), attributed to their abundant bioactive compounds. This review comprehensively illustrates the key mechanisms underlying the therapeutic potential of walnuts in IBD management, including the modulation of intestinal mucosa permeability, the regulation of inflammatory pathways (such as NF-kB, COX/COX2, MAPCK/MAPK, and iNOS/NOS), relieving oxidative stress, and the modulation of gut microbiota. Furthermore, we highlight walnut-derived anti-inflammatory compounds, such as polyunsaturated fatty acids (PUFA; e.g., ω-3 PUFA), tocopherols, phytosterols, sphingolipids, phospholipids, phenolic compounds, flavonoids, and tannins. We also discuss unique anti-inflammatory compounds such as peptides and polysaccharides, including their extraction and preparation methods. Our review provides a theoretical foundation for dietary walnut supplementation in IBD management and provides guidance for academia and industry. In future, research should focus on the targeted isolation and purification of walnut-derived anti-inflammatory compounds or optimizing extraction methods to enhance their yields, thereby helping the food industry to develop dietary supplements or walnut-derived functional foods tailored for IBD patients.

## 1. Introduction

The intestine is tasked with regulating tolerance to food and microbial antigens while combating pathogens. Loss of immune tolerance can lead to the inflammatory bowel diseases (IBDs) such as Crohn’s disease (CD) and ulcerative colitis (UC), significantly impacting patients’ quality of life, risk of hospitalization, complications, and likelihood of surgery [1]. Since 2000, the global incidence of IBDs has been increasing, currently affecting up to 1 in 200 individuals in Western countries [2]. It is generally accepted that genetic, immunological, and environmental factors (especially dietary factors) contribute to the risk of onset and progression of IBD [3]. The primary mechanisms underlying IBD include the modulation of intestinal mucosa permeability; the regulation of inflammatory pathways such as NF-kB, COX/COX2, MAPCK/MAPK, and iNOS/NOS; the alleviation of oxidative stress; and the modulation of gut microbiota composition and function [3]. The interaction between gut microbiota and dietary substrates in IBD has been extensively studied during the past decade. Of note, metabolites derived from the microbiota and food consumption can alter bile acid and short-chain fatty acid (SCFA) production, impact immune homeostasis, and affect the maintenance of mucosal integrity in the intestine [4]. On the other hand, the consumption of natural antioxidants, for instance, nuts, green tea, and legumes, can effectively defend against oxidative stress and regulate the inflammatory response in the gut, thereby contributing to the prevention of IBD development [5]. There is evidence that plant-based diets (which incorporate nuts) have a protective effect in IBD [6,7,8]. Hence, dietary management with nuts is emerging as a promising approach for attenuating IBD progression [9].

Walnuts (*Juglans regia* L.), a significant component of the Mediterranean and Asian diets, are increasingly becoming a staple across various dietary patterns worldwide due to their nutritional value and health benefits. The components of walnuts include the enrichment of omega-3 fatty acids, essential amino acids, vitamins (e.g., E, B6, B1), polyphenols, phytosterols, and other nutrients. Over the last few decades, an increasing amount of research has revealed the potential health benefits of dietary supplementation of walnuts, attributed to their abundance of antioxidant and anti-inflammatory bioactive content. Notably, dietary walnut supplementation [10] and walnut-derived bioactive compounds [11] have received renewed attention for their therapeutic potential in regulating IBD. For instance, dietary walnut supplementation has been shown to alter mucosal metabolite profiles during DSS-induced colonic ulceration, alleviating inflammatory intestinal tissue injury. Moreover, walnut oil, containing lipids and lipophilic bioactive compounds, has demonstrated regulatory effects on inflammatory pathways, such as TLR4/NF-κB pathways [12], and it has been shown to reduce oxidative stress in mice studies [13,14]. The regulation of microbiota by walnut oil supplementation in mice has also shown improvements in IBD. From these studies, it becomes evident that lipophilic components of walnuts, such as polyunsaturated fatty acids (PUFAs), phytosterols, tocopherols, and sterols, play a role in modulating IBD. On the other hand, the hydrophilic components of walnuts also exhibit potential protecting effects of IBD. For instance, walnut phenolic extract has been shown to inhibit NF-kB signaling and ameliorate colitis and colitis-associated colon cancer in mice [15]. Notably, in addition to these small-molecule bioactive compounds, large-molecule bioactive compounds such as walnut proteins, peptides, and polysaccharides are also being investigated for their potential in improving IBD. For example, walnut protein peptides have been found to ameliorate DSS-induced ulcerative colitis damage in mice [15]. It is also worth noting that while only the nuts of walnuts are typically consumed, other parts such as pomace, green shell, shell, skin, and leaves are often discarded or used as animal feed. In recent years, an increasing number of people have discovered that these by-products also contain a significant number of bioactive compounds [16]. Notably, more anti-inflammatory peptides have been discovered in walnuts and the by-products of walnuts [17]. Polysaccharides extracted from walnut green husk have been found to prevent colonic tissue damage in high-fat-diet-fed rats [18].

The current research increasingly demonstrates the regulatory effects of walnuts on IBD, providing a promising approach for the dietary treatment of IBD. In this review, we comprehensively summarize the possible underlying mechanisms of the regulating effects of walnuts on IBD and their pertinent anti-inflammatory compounds. By discussing the therapeutic potential of walnuts on IBD, we aim to guide future research and clinical applications, provide dietary advice for IBD patients, and offer suggestions for the food industry to develop functional foods for IBD patients.

## 2. The Regulating Effects and Mechanisms of Walnuts and Its Derived Bioactive Compounds’ Roles in Intestinal Inflammation

**Figure 1:**
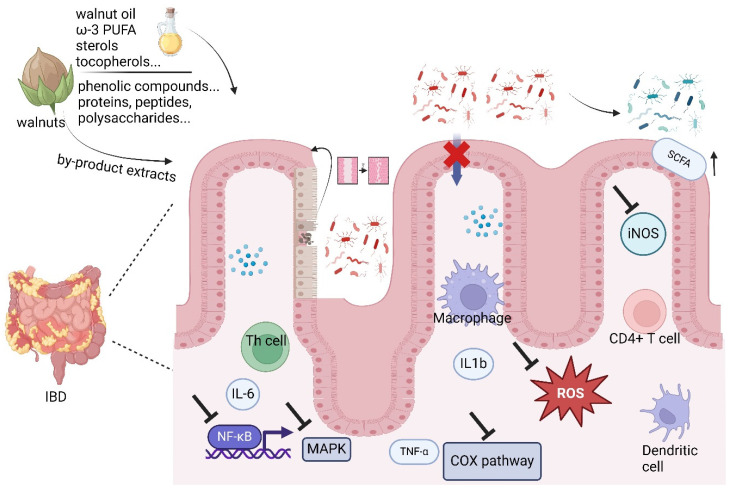
The mechanism of walnuts regulating IBD. (1) An illustration of the intestinal mucosal barrier and the effect of walnuts on permeability. (2) A depiction of the antioxidant effects of walnuts on ROS. (3) A pathway map showing NF-κB, COX/COX-2 and MAPK signaling modulation by walnuts. (4) Diagram showing changes in gut microbiota composition due to walnut consumption.

Walnuts, as a “super food”, contain abundant bioactive compounds, demonstrating significant therapeutic potential for IBD. The synergistic effects of these bioactive compounds in walnuts not only regulate various inflammatory pathways but also relieve oxidative stress within the digestive tract, improve dysregulated immune responses, and modulate gut microbiota (Figure 1).

### 2.1. Intestinal Mucosa Permeability

In the mucosal barrier of IBD patients, the changes in mucus layer composition and disruptions in adhesion molecules impact paracellular permeability. It has been suggested that nutrients released from nuts, especially walnuts, during digestion can improve the integrity of the cell wall structure, thus protecting the intestine barrier and improving the permeability of intestinal mucosa [19]. In an in vivo study by Bartoszek et al. [20], mice were randomly allocated to three groups and fed with either a standard diet containing 7% fat by weight or a diet containing either 7% sunflower oil or 7% walnut oil by weight for 8 weeks. Subsequently, 3% dextran sulfate sodium (DSS) was added to drinking water for 5 days to induce colitis. The results revealed that a diet enriched with walnut oil led to an improvement in the damage score within the inflamed colon, notably restoring ion transport and colonic wall permeability, compared to both sunflower oil-fed and the DSS-induced colitis groups. Notably, inflammation-induced alterations in the expression of tight junction proteins (TJs) and free fatty acid receptors (FFARs) were partially reversed by walnut oil [20]. Similarly, walnut oil has been found to improve the integrity of the mucosa in the duodena of Kunming healthy mice [13], suggesting that walnut oil could benefit IBD patients. Other components in walnuts have also been shown to protect the intestinal mucosa permeability. For instance, in high-fat-diet-fed rats, supplementation with walnut green husk polysaccharides (600 mg/kg·bw) was found to upregulate the expression of colonic tight junction proteins (zonula occluden-1 and occludin), preserving colonic barrier function in rats [18]. These studies highlight the role of walnuts in maintaining the integrity of the intestinal barrier in cases of IBD.

### 2.2. Oxidative Stress and ROS

In IBD, oxidative stress, an imbalance between the production and elimination of reactive oxygen species (ROS), occurs not only in the inflamed intestinal mucosa but also extends into the deeper layers of the intestinal wall, representing a characteristic and pathogenic factor of the disease [21]. One study revealed that DSS colitis mice supplemented with walnut oil (2.5 mL/kg·d for 27 days) exhibited a reduction in the production of ROS and release of pro-inflammatory cytokines. Additionally, walnut oil downregulated the expression of genes related to the NLRP3/ASC/Caspase-1 inflammatory pathway [22].

Recent evidence has demonstrated the potential antioxidative and anti-inflammatory advantages of walnut oil in IBD patients. As a traditional remedy and dietary supplement, walnut oil enhances enzyme activity, particularly superoxide dismutase (SOD) and glutathione peroxidase (GPx), thereby reducing oxidative stress and safeguarding the intestinal barrier [23,24]. In addition to the lipophilic components in walnut oil, the polar compounds in walnuts have shown activity in alleviating oxidative stress. In acetic acid-induced experimental colitis rats, treatment with 10 mg/kg walnut ethanol extract for 8 days effectively attenuated colonic damage scores and increased levels of total sulfhydryl (SH) groups, superoxide dismutase (SOD), and glutathione peroxidase (GPx) compared to the untreated colitis group [25]. In addition, juglone (JUG), a unique phenolic compound in walnut trees, has shown potent antioxidant and immunoregulatory activities [26]. Specifically JUG treatment (0.04 *w*/*v*) significantly mitigated body weight loss and the disease activity index in DSS colitis mice compared to controls. Additionally, JUG administration reversed the activation of mitochondrial uncoupling protein 2 and NF-kB p65, as well as the inhibition of kelch-like ECH-associated protein 1 and NF-E2-related factor 2 induced by DSS. Collectively, walnuts appear to confer antioxidative protection against gut tract damage induced by colitis.

### 2.3. NF-κB and Cytokines

As a master regulator of gene transcription, the activation of the NF-κB pathway plays a key role in the development of IBD and offers a promising treatment strategy for IBD patients [27]. The walnut oil phenolic extract and walnut peptides have been extensively studied for their ability to regulate the NF-κB pathway and downstream cytokines to promote intestinal healing in murine models of IBD [14,15,28]. For instance, in mice, the intragastric administration of 2.5 mL/kg walnut oil for 4 weeks decreased levels of tumor necrosis factor-α (TNF-α), interleukin-6 (IL-6), and IL-1β. Additionally, walnut oil (3 mg/kg) reduced the expression of key genes in the Toll-like receptor 4 (TLR4)/NF-κB pathway in acute jejunum injury induced by lipopolysaccharides (LPSs) [14]. The walnut-derived peptide LPLLR (LP-5) has also shown the ability to suppress the inflammatory NF-κB/MLCK/MLC signaling pathway activity in DSS colitis mice. Interestingly, the peptide also elicits neuroprotective effects by ameliorating hippocampal neuron damage and preserving blood–brain barrier integrity through the downregulation of microglia in colitis mice [28]. In both acute (4%) and chronic (2%) DSS colitis mouse models, mice administered walnut phenolic extract once daily via oral gavage (20 mg/kg) had attenuated TNF-α-induced IκB phosphorylation/degradation and NF-κB DNA binding activity. Walnut phenolic extract has also been shown to significantly reduce tumor development in a murine colitis-associated colon cancer (CAC) model. [15]. Urolithins, gut-microbiota-derived metabolites of ellagitannins, the most abundant phenolic compound in walnuts, were shown to inhibit LPS-induced inflammation in RAW 264.7 murine macrophages via NF-κB signaling pathways [29]. Juglone, a bioactive compound unique to walnuts, significantly attenuated colonic tissue damage and inflammation in mice, and it reduced NF-κB levels (*p* < 0.001) at an oral dosage of 150 mg/kg compared to controls [30].

### 2.4. COX/COX-2

COX-2 is induced in the epithelium of the large intestine during active IBD in humans and in the inflamed tissues of IL-10-deficient mice [31]. Conversely, COX-1/PGE2 serves as a protective mediator in UC [32]. In vitro studies have shown that walnut peptide, LPF, suppresses the mRNA expression of inducible nitric oxide synthase (iNOS), COX-2, and TNF-α in lipopolysaccharide-irritated RAW264.7 cells [33]. Additionally, emodin, a natural anthraquinone derived from walnut husks, has been shown to reduce COX-2 expression at both the mRNA and protein levels, thereby lowering the disease activity index in mice with DSS-induced acute colitis [34]. This suggests the potential anti-inflammatory role of walnuts in targeting the COX pathway in both in vitro and in vivo studies.

### 2.5. MAPCK/MAPK

Mitogen-activated protein kinases (MAPKs) are intracellular serine/threonine-specific kinases crucial for converting extracellular stimuli into various cellular processes [35,36], including the expression and activation of pro-inflammatory cytokines such as interferon (IFN)-γ, TNF-α, IL-1β, and IL-8, thus contributing to the pathogenesis of IBD [35,37]. Moreover, research has elucidated that walnut protein peptides (SHTLP, HYNLN, and LGTYP), as identified through virtual screening, ameliorate dysfunction of the intestinal mucosal barrier and mitigate inflammation by suppressing activation of the TLR4-MAPK pathway [38]. Further exploration is required to elucidate which constituents of the MAPK family, such as the extracellular signal-regulated kinases (ERKs), the c-Jun N-terminal kinases (JNKs), and the p38 MAPKs family, contribute most significantly to the anti-inflammatory pathways mediated by walnuts in IBD.

### 2.6. iNOS/NOS

In human IBD patients, the expression of inducible iNOS contributes to the initiation and maintenance of inflammation, suggesting a potential pro-inflammatory role of iNOS in the development of IBD [39,40,41]. An in vitro study showed that walnut ethyl acetate extract inhibits nitric oxide (NO) production in LPS-stimulated RAW 264.7 macrophages, indicating the anti-inflammatory function of walnuts in inflammatory diseases [42]. Moreover, the administration of emodin nanoparticles has been shown to suppress the expression of iNOS, COX2, and IL-1β at both the mRNA and protein levels, thereby preventing the damage caused by DSS-induced colitis [34]. However, recent research on experimental IBD animal models suggests that constitutive and inducible NO production may be beneficial during acute colitis [41]. Further murine studies are needed to determine the anti-inflammatory effects of walnuts by targeting iNOS signaling pathways.

### 2.7. Microbiome

Emerging studies have revealed that gut-microbiota-derived metabolites play crucial roles in maintaining intestinal homeostasis and modulating the progression of intestinal diseases, influencing both metabolic and immunological pathways [43,44,45]. As a dried fruit, walnuts have been extensively studied for their role in regulating gut microbiota in both murine and human studies. For instance, mice fed with walnut oil demonstrated a notable shift in gut microbiota composition, moving from a dominance of pathogenic bacteria, such as *Helicobacter*, towards an increase in probiotic populations. This dietary intervention also resulted in an increased immune organ index (spleen) and elevated levels of secretory immunoglobulin A (S-IgA) in the small intestine [13]. Previous studies have shown that S-IgA plays a critical role in regulating gut microbiota composition by inhibiting or eliminating pathogenic microorganisms while promoting the colonization of beneficial probiotics [46]. This indicates that walnut oil not only modulates gut microbiota towards a more favorable profile but also enhances gut immunity, contributing to improved intestinal health. In addition to the lipophilic bioactive compounds in walnut oil, in rats fed a high-fat diet, treatment with 750 mg/kg of body weight of walnut meal ethanol extracts rich in polyphenols significantly decreased the abundance of Gram-negative bacteria, particularly *Fusobacterium varium* and Bacteroides vulgatus, while markedly increasing the abundance of *Lactobacillus animalis*, as indicated by 16S rDNA sequencing [47]. Moreover, except for the edible part of walnuts, byproducts of walnut, such as walnut husk extract or leaf extract exhibit regulatory effects on the gut microbiome such as *Clostridium* spp. and *Faecalibacterium prausnitzii* in colonic diseases [48]. In high-fat-diet-induced colonic damage in rats, the consumption of walnut green husk polysaccharides improved dysbiosis in the gut microbiota by enhancing bacterial diversity and reducing the relative abundance of potentially pathogenic bacteria in the colon [18]. Moreover, juglone, a unique compound found in walnut trees and other plants, exhibits potent antimicrobial and immunoregulatory activities. Evidence indicates that juglone enriches beneficial microbes while not promoting pathogens such as *Escherichia* and *Shigella*, which were shown to predominate in a 3% DSS colitis mouse model [26]. In another DSS colitis (2.7%) study, juglone treatment enhanced the ratio of *Firmicutes* to *Bacteroidota* and increased the abundance of *Actinobacteriota* while decreasing *Verrucomicrobiota* abundance [49]. Collectively, walnut consumption appears to be a promising supplement for positively regulating gut microbiota in IBD.

### 2.8. Metabolic Markers

The levels of metabolites in the gut are associated with intestinal barrier functions, especially short-chain fatty acids (SCFAs) such as butyric acid and propionic acid [50]. In DSS colitis mice, walnuts induced significant increases in several PUFAs, including docosahexaenoic acid (DHA) and 9-oxo-10(E),12(E)-octadecadienoic acid (9-oxoODA), as well as kynurenic acid. In colon tissue samples, walnut consumption significantly elevated levels of S-adenosylhomocysteine (SAH) and betaine, critical components of fatty acid β-oxidation. These metabolite changes may contribute to the observed protection against DSS-induced inflammatory tissue injury [10]. In addition, protein–protein interaction (PPI) networks of amino acids and their metabolites in the Western pattern diet in high-fat-diet (HFD)-fed mice revealed significant remodeling effects of the walnut peptide on the intestinal flora’s abundance and diversity. This remodeling resulted in a reduced Firmicutes/Bacteroidetes (F/B) ratio, the repair of the intestinal mucosal barrier, alterations in the contents of SCFAs, and the alleviation of intestinal inflammation in HFD-fed mice [51]. Clinical studies have shown that a walnut-enriched diet increases SCFA production and improves the relative abundances of *Prevotellaceae* and *Allobaculum* in the gut. Specifically, in a randomized, controlled, prospective, cross-over clinical study conducted on 194 volunteers, eight weeks of walnut consumption (43 g/day) significantly increased *Ruminococcaceae* (*p* < 0.02), while *Clostridium* sp. cluster *XIVa* species (*Blautia; Anaerostipes*) decreased significantly (*p* < 0.05) [52]. Moreover, a 3-period, randomized, crossover, controlled-feeding study involving 35 participants suggested that following a walnut-enriched diet (57 g/day per 2100 kcal) for two weeks may increase the endogenous production of homoarginine, which plays a key role in creatine and energy metabolism and immune response. This effect is mediated through the gut microbiota-driven upregulation of glycine amidinotransferase [53]. In one randomized crossover study, 18 healthy men and women (mean age: 53.1 years; body mass index: 28.8 kg/m^2^) participated in two 3-week diet periods, receiving isocaloric diets containing either 0 or 42 g of walnuts per day, with a 1-week washout period between the diet phases. The results demonstrated that fecal secondary bile acids, specifically deoxycholic acid and lithocholic acid, were reduced by 25% and 45%, respectively, following the walnut treatment compared to the control treatment (*p* < 0.05). Additionally, serum low-density lipoprotein (LDL) cholesterol and the noncholesterol sterol campesterol concentrations were 7% and 6% lower, respectively, after walnut consumption compared to the control treatment (*p* < 0.01) [54]. These findings suggest a potential role for walnuts in regulating metabolism in IBD.

### 2.9. miRNA

Chronic inflammation in patients with IBD elevates the risk of colorectal cancer. During the past few decades, walnuts have garnered attention for their potential anti-tumor properties as a dietary supplement [15,55,56]. Emerging research indicates that microribonucleic acids (miRNAs) may elucidate the link between walnut consumption and reduced risk of colorectal neoplasia. Researchers have discovered that mice injected with HT-29 colon cancer cells and given a walnut-based diet for 25 days exhibited suppression of final tumor size. Walnut treated mice also had decreased expression of the miRNAs 1903, 467c, and 3068 (*p* < 0.05), along with an increase in the expression of miRNA 297a * (*p* = 0.0059) compared to controls. These findings indicate that walnut treatment can modulate miRNA expression, potentially affecting the transcripts of target genes involved in pathways associated with anti-inflammation, antivascularization, antiproliferation, and apoptosis in colonic cancer [57].

### 2.10. Summary

Taken together, walnuts, including walnut oil; extracts, especially phenolic extracts; peptides; and polysaccharide, as well as their by-products such as walnut husks, have demonstrated promising antioxidant, antimicrobial, and immunoregulatory effects via several key signaling pathways, including NF-κB, COX2, MAPK, and NOS, in both in vitro and in vivo studies. These studies have shown that components of walnuts can effectively protect the intestinal mucosal layer from damage and regulate intestinal permeability. Furthermore, walnuts have been shown to influence the gut microbiota composition by enhancing the ratio of *Firmicutes* to *Bacteroidota* and increasing the abundance of *Actinobacteriota*, while decreasing the abundance of potentially harmful microbiota. Despite these promising findings from animal models and in vitro studies, more research is still required. Further studies need to be performed in humans to confirm the benefits of walnut consumption. Specifically, clinical trials are needed to determine the efficacy and safety of walnut consumption in IBD patients. Such studies should aim to establish optimal dosages, identify potential side effects, and understand the long-term impacts of walnut intake on gut health and overall well-being in IBD patients.

In addition, current research on the potential of walnuts to regulate IBD is predominantly focused on mixtures such as walnut oil and extracts. Although these studies have shown promising results, the specific active compounds responsible for these beneficial effects remain unidentified. Further research is required to isolate and characterize the individual bioactive compounds within walnuts that contribute to their anti-inflammatory and immunoregulatory properties.

## 3. Anti-Inflammatory Components in Walnuts

Walnuts are abundant in nutrients and bioactive compounds, some of which have demonstrated anti-inflammatory effects, potentially contributing to the therapeutic effects of walnuts on IBD, including lipids and lipophilic bioactive compounds such as PUFAs; sphingolipids, and phospholipids; water-soluble constituents such as phenolic compounds, flavonoids, and tannins; and macromolecular components like proteins, peptides, and polysaccharide.

### 3.1. Lipids and Lipophilic Bioactive Compounds

Walnuts are known for their high lipid content (52–70%), which is mainly composed of polyunsaturated fatty acids (PUFAs), especially high in the ω-3:ω-6 ratio, which exceeds the ratios of other tree nuts [58]. Moreover, walnuts contain various lipophilic bioactive compounds including phytosterols, tocopherols, sphingolipids, and phospholipids. These lipid and lipophilic compounds are most commonly found in walnut oil, suggesting potential anti-inflammatory activity. Hence, in this section, we offer an overview of the health benefits, extraction methodologies, and medicinal properties associated with walnut lipids and lipophilic bioactive compounds, laying the groundwork for their potential use in IBD dietary management.

#### 3.1.1. Polyunsaturated Fatty Acids

Walnuts are abundant in PUFAs, including linoleic acid (C18:2), α-linolenic acid (C18:3), 5,11-eicosadienoic acid (C20:2), and arachidonic acid (C20:4), accounting for 69–72.8% of total fatty acids [58]. Compared to other plant oils, walnut oils contain more ω-3 PUFA α-linolenic acid (10–18%) and ω-9 monounsaturated fatty acid (MUFA) oleic acid (11.26–25.09%) (Table 1), endowing walnut oil with better anti-inflammatory activity [20]. Furthermore, if ω-3 PUFA supplies are adequately protected from oxidation, their post-ingestion anti-inflammatory activity will not be compromised. However, due to the higher PUFA contents of walnuts, they are more prone to oxidation, and these oxidized lipids, especially oxidized ω-6 PUFA, have been shown to increase the risk of developing IBD [59]. Therefore, preventing PUFA oxidation in walnut products will be a crucial step to preserve their anti-inflammatory activity, and the beneficial role of PUFA, especially ω-3 PUFA, in IBD has been extensively reviewed [60,61].

#### 3.1.2. Tocopherols

The anti-inflammatory activity of walnut lipid extracts may be attributed to not only to essential fatty acids but also to the abundance of tocopherols [77]. Total tocopherol concentrations in walnuts range from 359.2 to 420.6 mg/kg oil, with γ-tocopherol (315.3–351.2 mg/kg) being the major tocopherol, followed by α-tocopherol (25.5–40.3 mg/kg), then δ-tocopherol (16.3–25.1 mg/kg) and β-tocopherol (2.1–4.05 mg/kg) (Table 1) [62]. Tocopherols have been demonstrated as powerful lipid-soluble antioxidants that protect cell membrane lipids from oxidation, thus alleviating oxidative stress [78]. Furthermore, γ-tocopherol, the most abundant tocopherol in the lipid fraction of walnuts, can regulate signaling pathways related to inflammation such as NF-kB, and COX2 pathways [79], thus contributing to the attenuation of IBD. Of note, tocopherols also act as antioxidants to protect the PUFA in walnuts from oxidation. Therefore, the development of walnut products enriched with tocopherols, such as tocopherol-enriched walnut oil, could maximize the anti-inflammatory activity of ω-3 PUFA, ensuring that it is a suitable dietary choice for IBD patients.

#### 3.1.3. Phytosterols

In walnuts, phytosterols exist in various forms, including free sterols and conjugates such as sterol esters, sterol glycosides, and acylated sterol glycosides (Table 1). Zhang et al. quantified sterol contents in walnut oils from different cultivars, with the ranges of β-sitosterol, campesterol, and stigmasterol being determined to be 868.84–1385.18 mg/kg, 16.87–71.07 mg/kg, and 24.29–40.65 mg/kg, respectively. At least 16 sterol compounds have been reported in walnut oil, including cholesterol with a content of 8–38 mg/kg [80]; cholestanol, brassicasterol, and 24-methylenecholesterol B-sitosterol with a content of 772–2520 mg/kg [63]; B-sitosterol, brassicasterol, and campesterol with a content of 66.19–111.75 mg/kg [64]; campestanol and stigmasterol with a content of 13.49–27.31 mg/kg [64]; clerosterol with a content of 11–50 mg/kg [63]; and ∆5,23-stigmastadienol, ∆5,24-stigmastadienol, ∆5-avenasterol ∆7-stigmastenol, and ∆7-avenasterol with a content of 78.98–141.42 mg/kg [64]. Notably, β-sitosterol was identified as the predominant sterol in walnut oil, constituting over 90% of the total sterol content [81]. Phytosterols have been reported to exhibit a variety of physiologically active effects, for example, stigmasterol restores Treg/Th17 cell homeostasis through butyrate-mediated PPARγ activation, thereby attenuating IBD [82], while β-sitosterol may ameliorate colitis by regulating the NF-κB pathway [83,84]. Therefore, optimizing extraction methods for phytosterols from walnuts is crucial for fully exploiting their anti-inflammatory properties, particularly for obtaining sterols such as stigmasterol and β-sitosterol. For example, Feng et al. investigated direct hydrolysis extraction with citric acid to optimize sterol extraction from walnut husk, and the phytosterols obtained were 912.452 ± 17.452 μg/g dry weight at pH 2.0 and 55.81 °C with a solid/liquid ratio of 17.12, which is more effective and environmentally friendly than the traditional solvent extraction method [85]. In addition, such attempts have confirmed the potential of extracting phytosterols from walnut by-products, such as husks. Future research should focus on improving these extraction techniques and exploring the full therapeutic potential of walnut-derived phytosterols.

#### 3.1.4. Sphingolipids

Walnuts contain three types of sphingolipids (Table 1): ceramides, glycosphingolipids, and hexosylceramides; these components constitute 24.55% of the total lipids, approximately 30 µg/mL [65]. The sphingolipid content in walnut oil has been measured as 2500 ± 173 mg/kg oil in previous studies [66]. Phytoceramides, a class of sphingolipids, have been reported to ameliorate Alzheimer’s disease-associated cognitive deficits, potentially by inhibiting p-tau formation through anti-apoptotic and anti-inflammatory activities, as well as by promoting the PI3K/Akt/CREB signaling pathways [Phytoceramide ameliorates ß-amyloid-protein-induced memory impairment and neuronal death in mice]. However, the effects of sphingolipids in walnuts on IBD, inflammation, and oxidative stress remain to be studied.

#### 3.1.5. Phospholipids

Phospholipids have been comprehensively identified in walnuts, including phosphatidylcholine (PC), phosphatidylethanolamine (PE), phosphatidylglycerol (PG), phosphatidylinositol (PI), phosphatidylserine (PS), lysophosphatidylcholine (LPC), and lysophosphatidylethanolamine (LPE) (Table 1) [67]. Song et al., developed a mass spectrometry (HILIC-ESI-IT-TOF-MS) method to systematically characterize the phospholipid profile in walnuts, and 96 phospholipid molecules were detected in walnuts. Notably, the contents of PG (34:2), PE (34:2), PE (36:4), PI (34:2), and PC (34:2) in walnut oil were quantified at 304.4 μg/mL, 1713.7 μg/mL, 1023.5 μg/mL, 2164 μg/mL, and 1103.4 μg/mL, respectively. Compared with other nuts, walnuts are a favorable nut resource with diverse and higher phospholipid contents [86]. The phospholipids in walnut oil may contribute to its therapeutic activity in treating IBD. Despite this potential, the specific impacts of walnut phospholipids on IBD, inflammation, and oxidative stress require further investigation.

### 3.2. Phenolic Compounds

Walnut polyphenolic compounds, which mainly include phenolic acids, flavonoids, and tannins, have powerful anti-inflammatory, antioxidant, and free radical scavenging properties. In addition, these polyphenol compounds are not only found in the edible kernels of walnuts but are also abundant in other parts of walnuts, such as pomace, green husk, shell, pericarp, and leaves. In this section, we provide an overview of these polyphenolic compounds found in various parts of walnuts, particularly those unique compounds exclusive to walnuts, and their potential anti-inflammatory activities (Table 1).

#### 3.2.1. Phenolic Acids

Over 120 phenolic compounds have been identified in walnuts [87], with ellagic acid, caffeine acids, chlorogenic acid, neochlorogenic acids, cinnamic acids, 3-p-coumaroylquinic acid, and 4-p-coumaroylquinic acid being the most abundant. Cinnamic acid, in particular, exhibits the highest content in walnut kernel [68]. These phenolic acids in walnuts exhibit significant antioxidant activity, having the potential to ameliorate IBD by alleviating oxidative stress. Piwowarski et al.’s study showed that ellagitannins, which are metabolites of gut microbiota, can effectively reduce the expression of IL-1 β, TNF-α, and IL-6 mRNA, important for improving intestinal inflammation [88]. In addition, polyphenolic compounds in walnuts have been shown to have strong antioxidant activity in vitro antioxidant studies, and their antioxidant capacity is comparable to those of VC and BHA within a certain range, which has potential value in the study of inflammatory diseases caused by oxidative stress [89]. Of note, the extraction method, time of harvest, and geographical location of the walnut harvest influence phenolic acid content [70].

#### 3.2.2. Flavonoids

Walnuts contain ~17 different anti-inflammatory flavonoids [90], with 7-hydroxymethylcoumarin being the most abundant (245.3 mg/g) [69]. High flavonoid levels are also present in walnut pellicles, flowers, kernels, and husks. Although there is no direct evidence to prove the important role of flavonoids in walnuts in enteritis, multiple studies have shown this possibility. Yan et al.’s research has shown that flavonoids can effectively reduce the expression of NF-κ B and inflammatory factors in mice with radiation-induced enteritis, playing an important role in the treatment of radiation-induced enteritis [91]. In addition, Su et al.’s study showed that flavonoids can effectively improve tissue pathology and levels of inflammatory factors in mouse colitis models, effectively treating and preventing the occurrence of colitis [92]. Therefore, the potential therapeutic activity of flavonoids in walnuts for enteritis deserves further investigation.

#### 3.2.3. Tannins

Tannins are present in various parts of the walnut, with at least ten polyphenols identified in the fruit pellicle extract of English walnuts. The main tannin components, which are classified as hydrolyzable tannins, include monomeric ellagic acid, methyl gallate, and gallic acid in polymer form [93]. They are also the main phenolic substance in the walnut pellicles [94], including HHDP-glucose Isomer (259.17 µg/g) and bis-HHDP-glucose (349.44 µg/g) [70,71]. Among these, pedunculagin, an ellagitannin, is found in the highest concentration. It has been demonstrated that the BuOH fraction yields the highest content of pedunculagin [95]. Upon ingestion, pedunculagin converts into ellagic acid, which is further metabolized by intestinal microbiota into the bioactive anti-inflammatory urolithin. Several studies have shown that ellagic acid exerts its anti-inflammatory activity by inhibiting the expression of TNF-α, NF-κB, IL-6, IL-1β, iNOS, and COX-2 [96,97]. Numerous studies have demonstrated that walnuts exhibit greater antioxidant activity than other nuts due to hydrolyzable tannins [98]. Although there have been some studies on the anti-inflammatory and antioxidant effects of tannins in walnuts in recent years, the mechanism of this substance’s impact on intestinal inflammation needs further investigation.

#### 3.2.4. Junglone

Juglone is a small molecule compound of the naphthoquinone class, uniquely derived from walnuts. It is widely distributed in the leaves, roots, shells, fruits, and bark of walnuts, particularly in the green skin, which contains 283.4 µg/mg of juglone [72]. In recent years, juglone has been studied for its antioxidant, anti-tumor, antibacterial, and anti-inflammatory properties, particularly in the prevention and treatment of colitis. Chen et al. reported that juglone effectively ameliorates colon injury in mice by modulating inflammatory cytokines and oxidative stress, thereby preventing and treating colitis [26]. Similarly, Cui et al. developed juglone F127/TPGS mixed micelles using a thin-film dispersion method. In vivo studies have shown that these micelles effectively release juglone, significantly improving colitis symptoms in mice [99]. Moreover, Lu et al. demonstrated that juglone possesses potential therapeutic and preventive effects against colon cancer and associated inflammation [100]. Although numerous studies have highlighted the medicinal value of juglone in colitis, current research remains limited, and further investigation into the target genes or receptors of juglone is needed.

### 3.3. Proteins and Peptides

Walnut protein, comprising 14% to 28% of total protein content and containing 18 amino acids (including 8 essential amino acids), demonstrates high nutritional value and bioactivity [101,102,103]. Numerous studies have demonstrated the antioxidant, anti-cancer, immune modulation, and anti-inflammation effects of walnut peptides derived from the enzymatic hydrolysis of walnut proteins [104]. Therefore, in this section, we summarize the recent research on walnut proteins and peptides (Table 1) and discuss their great significance in anti-inflammatory activity, thus providing a valuable theoretical basis for studying the potential of treating IBD with walnut proteins and peptides.

#### 3.3.1. Proteins and Protein Hydrolyses

Walnut-derived proteins comprise albumin, globulin, protoprotein, and gluten [73], with essential amino acid content being 26.98%. These proteins have demonstrated their anti-inflammatory effects by modulating the NF-κB and COX pathways [105,106]. In mouse models of colitis, both walnut protein and its enzymatic products effectively suppressed serum IL-1β, IL-6, TNF-α levels, and myeloperoxidase (MPO) activity, thereby preventing colitis progression [73]. Wang et al. demonstrated effective antioxidant activity and antihypertensive ability in in vivo experiments on hypertensive rats using the enzymatic products obtained from the hydrolysis of walnut protein by alkaline protease and trypsin [107]. Similarly, this antioxidant activity was also demonstrated in the study by Li et al. [108].

#### 3.3.2. Bioactive Peptides

To date, by adjusting the different conditions and types of proteases involved in enzymatic hydrolysis, researchers have discovered an increasing number of active peptides derived from walnuts, particularly those with important anti-inflammatory value.

Wang et al. discovered several small peptides, such as LPF, GVYY, and APTLW, from walnut protease hydrolysates. These peptides, characterized by hydrophobic and aromatic amino acid residues, exert anti-inflammatory effects by downregulating inflammation-related enzyme activity and mRNA expression, thereby reducing pro-inflammatory mediators and cytokines [74]. In another study, mice fed walnut-derived low-molecular-weight peptide leucine–proline–phenylalanine was protective of dextran sulfate sodium (DSS)-induced colitis in mice [109]. Li et al. hydrolyzed walnut protein with alkaline protease and identified the active peptides RLWPF and VLRLF. In vivo experiments showed that they can regulate oxidative stress and inflammation by regulating the balance of gut microbiota [110]. In addition to enzymatic preparation of peptides, more novel methods have been developed. Notably, Xia et al. identified peptides (IPAGTPVYLINR, FQGQLPR, and VVYVLR) from walnuts, which have been shown to effectively reduce the expression of inflammatory factors and exhibit significant anti-inflammatory activity in a normal human colon mucosal epithelial NCM460 cell inflammation model induced by lipopolysaccharide (LPS), demonstrating potential value in the treatment of colitis [111]. In addition, Wen et al. utilized network pharmacology and molecular docking studies to demonstrate that glutathione may be the main active ingredient in walnut peptides, with potential value and activity in reducing inflammation and regulating gut microbiota and metabolites [51].

It is also worth mentioning that walnut residue is one of the main by-products of walnut processing. There are reports that anti-inflammatory peptides have been found in walnuts and their by-products [112], offering significant insights for future research on the anti-inflammatory activity of walnut by-products.

### 3.4. Polysaccharides

A walnut’s kernel, shell, and husk all hold significant concentrations of polysaccharides (2.48% to 5.40%), including xylose, trehalose, and mannose [113]. Walnut polysaccharides have been shown to inhibit inflammation by enhancing phagocytosis and stimulating the production of NO, tumor necrosis factor-α (TNF-α), and interleukins (IL-6 and IL-1β) in HepG2 and BGC-82 cell lines [75]. In addition, the walnut green peel comprising galacturonic acid (69.47%), galactose (11.18%), rhamnose (8.67%), arabinose (3.96%), glucose (2.21%), glucuronic acid (2.28%), xylose (0.83%), fucose (0.81%), fucose (0.59%), and mannose (0.59%)) has been shown to induce macrophage activation through the MAPK and PI3K/Akt pathways [114]. One study in rats found that walnut green husk was protective against HF-induced colonic tissue injury by improving intestinal microbiota composition, protecting gut barrier function [18].

## 4. Discussion

In recent years, there has been a growing body of research on the ameliorating effects of walnuts on IBD. Plenty of in vivo and animal studies have demonstrated that walnuts and their derived bioactive compounds improve inflammation in intestinal chronic conditions such as IBD by modulating inflammatory pathways such as the NF-κB, COX/COX-2, MAPCK/MAPK, and iNOS/NOS pathways while also alleviating oxidative stress and regulating the gut microbiome and gut metabolism. In addition to their well-known abundance of PUFAs, especially ω-3 PUFA, tocopherols, sterols, polyphenols, and flavonoids, more unique anti-inflammatory compounds have been identified from walnuts, such as peptides and polysaccharides. The current research remains promising; however, more focused pre-clinical studies and clinical studies are needed to exactly derive the mechanisms responsible for the roles that walnut bioactive compounds play in protecting the intestine in intestinal chronic conditions such as IBD.

We have highlighted the significant role of walnut-derived active compounds for the prevention of and improving inflammation in intestinal chronic conditions such as IBD, underpinned by potential molecular mechanisms. Bioactive compounds in walnuts are shown to prevent and treat IBD by regulating ROS levels, inhibiting oxidative stress responses, and potentially modulating the NLRP3/ASC/Caspase-1 inflammatory pathway. For instance, walnut oil has been shown to alleviate DSS-induced colitis in mice by inhibiting NLRP3 inflammasome activation and regulating gut microbiota [22]. Additionally, a primary regulatory factor for gene transcription has been identified as a key signaling pathway through which walnut peptides exert anti-inflammatory effects, thereby preventing intestinal inflammation and related diseases [28]. Other signaling pathways, including the COX/COX-2, iNOS/NOS, and MAPK/MAPK pathways, are also confirmed as principal mechanisms through which walnut bioactive compounds improve inflammation in intestinal chronic conditions such as IBD [33,34,38]. It is worth noting that bioactive compounds in walnuts have been confirmed to regulate the level of miRNA in vivo, thereby achieving regulatory control of target gene expression [57]. Interestingly, research has demonstrated that plant-derived miRNAs can be absorbed by the intestine and directly regulate gene expression in humans [76]. Whether miRNAs derived from walnuts can also be absorbed by the intestine to regulate inflammatory genes in the human body remains to be further discovered confirmed; this would represent a groundbreaking discovery in the field of nutrition and inflammation. At present, research on the potential of walnuts to regulate IBD mainly focuses on mixtures of walnut oil and extracts. Although these studies have shown promising results, the specific active compounds responsible for these beneficial effects are still unclear. Further research is needed to isolate and characterize individual bioactive compounds in walnuts that contribute to their anti-inflammatory and immunomodulatory properties, such as peptides with specific sequences, characterized polyphenolic compounds, and polysaccharides. Nevertheless, the molecular mechanisms underlying the effects of walnut bioactive compounds on IBD are complex and warrant further exploration. Understanding these mechanisms will provide a crucial theoretical foundation for the pharmacological study and therapeutic application of walnuts.

While it is interesting that the bioactive substances extracted from walnuts may not play a single role in the process of exerting their biological activity [115], synergistic interactions between various substances are increasingly being investigated, which may provide a new perspective for studying the molecular mechanisms of walnut bioactives that drive inflammation prevention. Walnuts contain a variety of anti-inflammatory active constituents (e.g., polyunsaturated fatty acids, tocopherols, phospholipids, tannins, peptides and polysaccharides) with potential health benefits. Although preliminary studies on the anti-inflammatory and antioxidant mechanisms of action of certain components have been conducted, there are still many functional components that need to be further explored, such as sphingolipids and phospholipids. In addition, the gastrointestinal absorption kinetics of active substances are often overlooked, for instance, pedunculagin converts into ellagic acid (EA) further metabolized by intestinal microbiota [96,97]; hence, improving the absorption of bioactive compounds such as pedunculagin in walnuts through carriers has important value in clinical medicine. In addition, determining the efficacy and safety of consuming walnuts in IBD patients through clinical trials is something to which we should pay attention. This type of study should aim to determine the optimal dosage, identify potential side effects, and understand the long-term effects of walnut intake on the intestinal and overall health of IBD patients.

Beyond the edible parts of walnuts, their by-products also offer significant potential. The walnut green husk is the primary walnut processing by-product, which has been lacking re-utilization for decades. In recent years, emerging studies have been applied to extract and identify the bioactive compounds of walnut green husk [116], especially polysaccharide [117] and phenolic compounds [118]. In addition to walnut green husk, dried walnut shells account for 50% of walnuts, which are often burned as fuel. However, researchers have found more than 30 phenolic compounds, including phenols, ellagic acid derivatives, and flavonoids, in the ethanolic extract of dried walnut shells [119]. Hence, more sustainable and efficient approaches need to be developed to utilize the by-products of walnut processing to avoid waste and develop more functional products from walnuts. In addition, different extraction methods can also influence the amounts and varieties of the extracted compounds. Hence, more processing methods can be optimized to make full use of walnut by-products, thus maximizing their health effects. In addition to the health benefits, there is a preliminary attempt to apply the walnut green husk extract to the packaging material of fresh-eating walnuts and extend their shelf lives [120]. The exploration of such applications aligns with the broader goal of reducing waste and maximizing the health benefits derived from walnuts by-products.

The diverse lipid and bioactive profiles of walnuts demonstrate their improving potential for inflammation in intestinal chronic conditions such as IBD. Future research should focus on optimizing extraction methods and exploring the full medicinal potential of walnut compounds. By doing so, we can fully harness the health benefits of walnuts, making significant strides in both clinical and pharmaceutical fields. In addition, the variability in the contents of these phytochemicals has been attributed to factors such as cultivar type [77], growth conditions including temperature and humidity [62], and the harvesting season [121]. Thus, future research endeavors may focus on optimizing cultivars and cultivation conditions to yield walnuts enriched with higher contents of anti-inflammatory bioactive compounds. Furthermore, exploring methods to optimize the extraction of phenolic compounds from walnuts and their by-products, as well as evaluating the anti-inflammatory activities of newly discovered compounds, would be a promising direction for future research on IBD’s dietary management with walnuts.

## 5. Conclusions

Dietary supplementation with walnuts has demonstrated efficacy in the inflammation of intestinal chronic conditions such as IBD, suggesting a promising approach for the dietary management of IBD. In this review, we have elucidated the underlying mechanisms of walnuts and derived bioactive compounds in regulating and improving IBD, summarizing the bioactive compounds with anti-inflammatory properties. However, current research largely remains at the stage of in vivo or animal studies, with limited clinical studies. Furthermore, while various mechanisms have been explored, most research focuses on mixtures or extracts such as walnut oil or walnut extracts, and further investigation is needed to identify the specific bioactive compounds targeting IBD regulation. In addition, research on anti-inflammatory compounds primarily focuses on the main edible part of walnuts, its kernels, while the abundant anti-inflammatory compounds in other by-products such as pomace, green shell, shell, skin, and leaves remain largely unexplored. In future research, achieving a comprehensive understanding of the chemical basis of the anti-inflammatory effects of walnuts requires that researchers not only explore more anti-inflammatory compounds from walnuts and their by-products but also establish the correlation between these anti-inflammatory compounds and their underlying mechanisms, thereby suggesting optimized processing methods and novel applications for food industries to develop functional walnut products with therapeutic potential for IBD patients.

## Figures and Tables

**Table 1:** The main anti-inflammatory active ingredients and contents in walnuts.

Anti-Inflammatory Classification	Compound Name		Content	References
Lipids and lipophilic bioactive compounds	Polyunsaturated fatty acids	ω-3 PUFA α-linolenic acid	10–18%	[20]
		ω-9 monounsaturated fatty acid (MUFA) oleic acid	11.26–25.09%	
	Tocopherols	γ-tocopherol	315.3–351.2 mg/kg	[62]
		α-tocopherol	25.5–40.3 mg/kg	
		δ-tocopherol	16.3–25.1 mg/kg	
		β-tocopherol	2.1–4.05 mg/kg	
	Phytosterols	β-sitosterol	868.84–1385.18 mg/kg	[63,64]
		campesterol	16.87–71.07 mg/kg	
		stigmasterol	24.29–40.65 mg/kg	
	Sphingolipids	ceramides	-	[65,66]
		glycosphingolipids	-	
		hexosylceramides	-	
	Phospholipids	phosphatidylcholine	PC (34:2) 1103.4 μg/mL	[67]
		phosphatidylethanolamine	PE (34:2) 1713.7 μg/ML PE (36:4) 1023.5 μg/mL	
		phosphatidylglycerol	PG (34:2) 304.4 μg/mL	
		phosphatidylinositol	PI (34:2) 2164 μg/mL	
		phosphatidylserine	-	
		lysophosphatidylcholine	-	
		lysophosphatidylethanolamine	-	
Phenolic compounds	Phenolic acids	cinnamic acid	213.38 µg/g	[68]
		tannic acid	312.57 µg/g, 1023.9 µg/g	
	Flavonoids	7-hydroxymethylcoumarin	245.3 mg/g	[69]
		eugenol	165.7 µg/g	
		apigenin	74.21 µg/g	
		catechin	44.21 µg/g	
	Tannins	HHDP-glucose Isomer	259.17 µg/g	[70,71]
		bis-HHDP-glucose	349.44 µg/g	
	Junglone	juglone	283.4 µg/mg	[72]
Proteins and peptides	Proteins and protein hydrolyses	albumin		[73]
		globulin		
		protoprotein		
		gluten		
	Bioactive peptides	LPF		[74]
		GVYY		
		APTLW		
Polysaccharides	Polysaccharides	xylose, trehalose, and mannose		[75,76]
		rhamnose, arabinose, galactose, glucose, xylose, and galacturonic acid	6.7%:16.5%:28.3%:11.2%:12.5%:24.8%	
		galacturonic acid, galactose, rhamnose, arabinose, glucose, glucuronic acid, xylose, fucose, fucose, and mannose	69.47%:11.18%:8.67%:3.96%:2.21% :2.28%:0.83%:0.81%:0.59%:0.59%	

## Data Availability

No new data were created or analyzed in this review. Data sharing is not applicable to this review article.
